# A statistical modeling approach based on the small-scale field trial and meteorological data for preliminary prediction of the impact of low temperature on *Eucalyptus globulus* trees

**DOI:** 10.1038/s41598-023-37038-8

**Published:** 2023-06-22

**Authors:** Tomoaki Chubachi, Taichi Oguchi, Kazuki Morita, Nanami Hayashi, Akira Kikuchi, Kazuo N. Watanabe

**Affiliations:** 1 Graduate School of Science and Technology, University of Tsukuba, Tsukuba, Ibaraki 305-8572 Japan; 2 Institute of Life and Environmental Sciences, University of Tsukuba, Tsukuba, Ibaraki 305-8572 Japan; 3 Tsukuba Plant Innovation Research Center, University of Tsukuba, Gene Research Center Bldg., Ten-Nodai, Tsukuba, Ibaraki 305-8572 Japan; 4 Graduate School of Life and Environmental Sciences, University of Tsukuba, Tsukuba, Ibaraki 305-8572 Japan

**Keywords:** Plant biotechnology, Plant stress responses, Statistical methods

## Abstract

*Eucalyptus* trees are important for industrial forestry plantations because of their high potential for biomass production, but their susceptibility to damage at low temperatures restricts their plantation areas. In this study, a 6-year field trial of *Eucalyptus globulus* was conducted in Tsukuba, Japan, which is the northernmost reach of *Eucalyptus* plantations, and leaf damage was quantitatively monitored over four of six winters. Leaf photosynthetic quantum yield (QY) levels, an indicator of cold stress-induced damage, fluctuated synchronously with temperature in the winters. We performed a maximum likelihood estimation of the regression model explaining leaf QY using training data subsets for the first 3 years. The resulting model explained QY by the number of days when the daily maximum temperature was below 9.5 °C over approximately the last 7 weeks as an explanatory variable. The correlation coefficient and coefficient of determination of prediction by the model between the predicted and observed values were 0.84 and 0.70, respectively. The model was then used to perform two kinds of simulations. Geographical simulations of potential *Eucalyptus* plantation areas using global meteorological data from more than 5,000 locations around the world successfully predicted an area that generally agreed with the global *Eucalyptus* plantation distribution reported previously. Another simulation based on meteorological data of the past 70 years suggested that global warming will increase the potential *E. globulus* plantation area in Japan approximately 1.5-fold over the next 70 years. These results suggest that the model developed herein would be applicable to preliminary predictions of *E. globulus* cold damage in the field.

In recent years, global demand for wood resources has been increasing along with the general movement away from fossil resources and toward sustainable economic activities^1^. As for plant-derived biomass, maize and sugar cane are already in practical use as materials for bioethanol fuel^2,3^, and wood biomass is also expected to be used in this capacity due to the large reserves of wood biomass and the fact that it does not compete with food production^2,4^. Plant-derived biomass has the potential to contribute additional advantages such as carbon neutrality and biodegradability^2^. Moreover, in addition to providing biomass resources for industries, forests also play an important role as carbon dioxide sinks in the carbon cycle^5^. Despite these many incentives for forest expansion, however, total forest area decreased rapidly by about 290 million hectares over the same period^6^. Such deforestation has been suggested to undermine climate change-mitigation efforts while also weakening ecosystems and promoting the emergence of novel epidemics, making it an urgent matter to curb deforestation and restore forests^7,8^.

The genus *Eucalyptus* is mostly indigenous to Australia and its surroundings but has been commercially introduced to tropical and subtropical regions of the world since the nineteenth century^9^. It has various advantages for cultivation, such as short rotation intervals, high biomass productivity, latent bud renewal, and tolerance to slightly saline soils, and is being cultivated in plantations worldwide^10^. The cultivated product has many applications, including use in pulp, building materials, oils, insect repellents, and aromatherapy^11^. Trees of the genus *Eucalyptus* are fast-growing evergreen broadleaf forest trees with higher growth potential and a faster growth rate than temperate forest trees, even in temperate zones^10^. However, *Eucalyptus* trees are also highly sensitive to low temperatures and rapid temperature change, and often die over cold winters even in temperate zones, although there are variations within and among species^12^. Susceptibility to low-temperature damage is thus a barrier to the introduction of *Eucalyptus* trees in some areas.

Among plantation trees in the *Eucalyptus* genus, the species *E. globulus* can produce white, very high-performance pulp with low residual lignin content and low degradation of cellulose and hemicellulose and is widely used in warm climates^13,14^. Thus, a reliable method for evaluating and predicting low-temperature damage could be critical to the management of *Eucalyptus* plantations or the genetic improvement of *Eucalyptus* plantation trees. A focus on plantation border regions, where cold damage tends to be more pronounced, would be particularly helpful. Nonetheless, there have been no long-term observation studies focused on cold damage to *E. globulus* in the borders of potential plantations of the genus *Eucalyptus*. In the present study, we conducted monitoring observations of cold damage on leaves of *E. globulus* over four separate winter seasons (each, November to March) in an experimental field in Tsukuba, Ibaraki, Japan, which is located within the northern border region of a *Eucalyptus* plantation.

The discovery of simple mathematical formulas inherent in complex life phenomena can provide clues to a fuller understanding of the phenomena themselves. Over the last several decades, developments in computers and statistical methods have made it much easier to uncover such formulas from the experimental observation of biological phenomena. And by using the found mathematical formula, it is possible to predict life phenomena at times and places that were once difficult to observe. For example, these approaches have yielded mathematical formulas for the prediction of growth zones, gene expression levels, and crop yields based on limited information such as temperature^15–19^. This approach—i.e., the development of a statistical model from data collected by observation over a limited time and region, followed by simulation based on the model—has the potential to expand the use of experimental data to times and regions where no observations are currently being made. In this study, we applied this approach to elucidation of the relationship between weather conditions and cold damage of *E. globulus* leaves based on our experimentally observed data and obtained an equation governing the association between photosynthetic quantum yield values of *E. globulus* leaves and temperature. Then, in order to verify the performance of the mathematical model, we simulated the potential for establishing plantation-based cultivations of *E. globulus* based on publicly available temperature data from around the world and compared it with the distribution of actual *Eucalyptus* plantations. In addition, we attempted to predict potential future planting sites based on trends in the potential for plantations of *E. globulus* predicted from temperature data over the past 70 years in 101 locations in Japan.

## Results

### Consideration of appropriate indicators for assessing leaf damage caused by low temperature in the field

**Figure 1:**
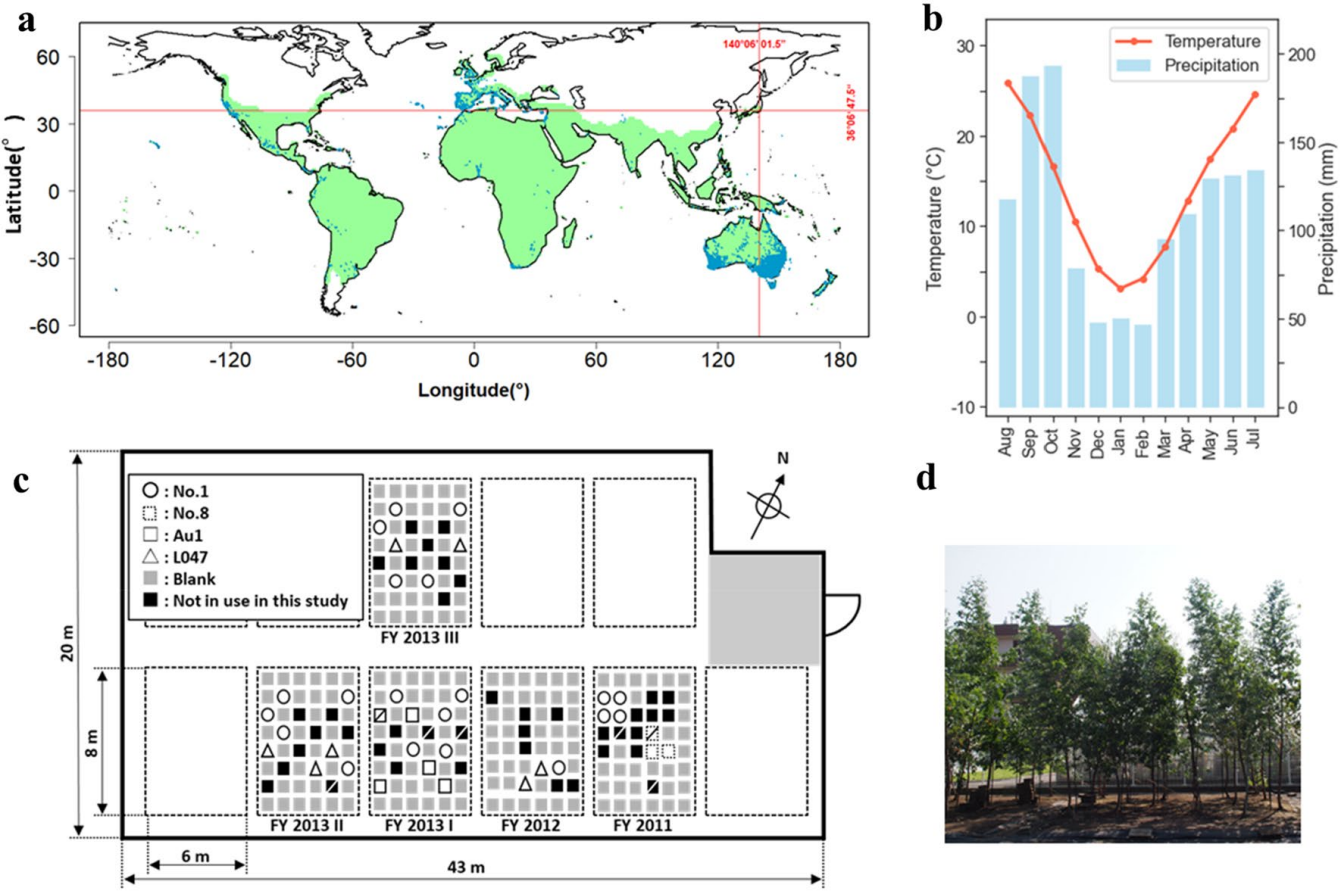
Location and plot design of the field trial to evaluate the cold sensitivities of *E. globulus* (**a**) Location of the field trial site. The red lines intersect at the isolated field where the trial was conducted at the Tsukuba Plant Innovation Research Center, University of Tsukuba (36°06 ′47.5 ″N 140°06′01.5″E). Light-green areas illustrate the global *Eucalyptus* cultivation areas based on FAO reports. Blue plots indicate sites of observed *E. globulus* and other *Eucalyptus* spp. cultivation reported on the GBIF database (https://www.gbif.org). The based map was drawn with ‘map’ package of R software under GPL2 licenses. (**b**) Monthly averages of daily mean temperature and monthly cumulative precipitation in Tsukuba for the past 30 years. (**c**) Plot design of the field trial. (**d**) A photo of the field trial site. The photo, taken in October 2015, shows *E. globulus* trees 2 years after being planted in the field.

**Figure 2:**
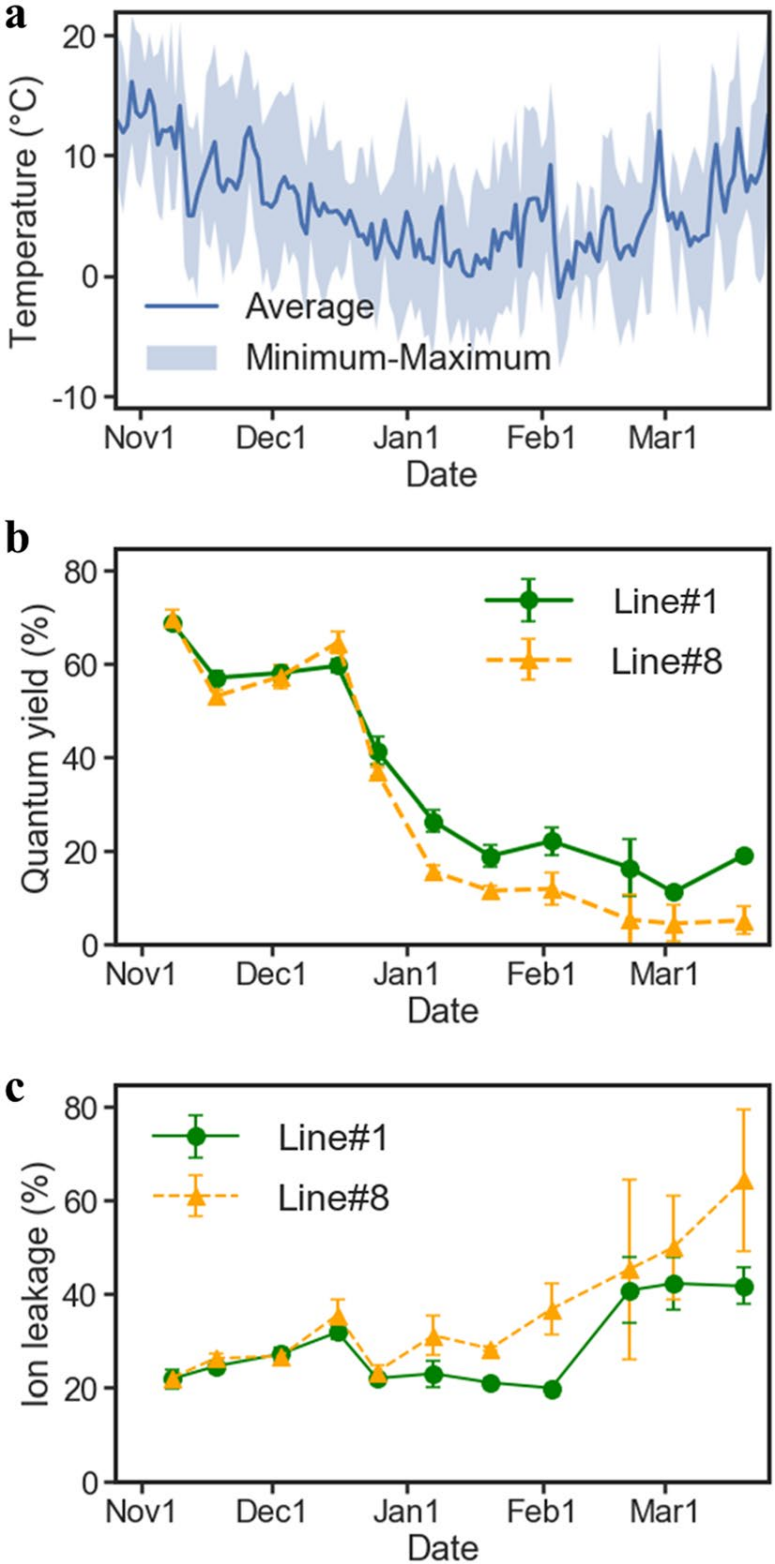
Temperature trends at the field trial site and QY and IL of *Eucalyptus* leaves in the second year during the period from November 2013 to May 2014 (**a**) Temperature trends at the field trial site. The daily average temperatures and the range between the daily maximum and daily minimum temperatures observed at the Center for Research in Isotopes and Environmental Dynamics, University of Tsukuba, are shown as a solid line and filled area, respectively. (**b**, **c**) Trends of leaf QY and IL levels. QY and IL observed in leaves of two genetically independent clonal lines of *E. globulus*, i.e., No. 1 and No. 8, during the period from November 2013 to May 2014 are shown in (**b**) and (**c**), respectively. Error bars indicate the intervals of standard errors.

In this study, we conducted a 6-year field trial of *E. globulus* in Tsukuba, Japan, which is located within the northern border region of an *Eucalyptus* plantation-based cultivation (Fig. 1a). To evaluate and compare the low-temperature sensitivities of field-grown *Eucalyptus* trees, we considered the leaf photosynthetic quantum yield (QY) and ion leakage (IL) as indices of as indices of low-temperature sensitivity. A preliminary experiment was conducted to monitor the QY and IL of leaves of field-grown *Eucalyptus* trees every 2 weeks from November 2013 through April 2014. On the site where the field trial was conducted, days with temperatures below 0 °C began to occur in early December in general, and temperatures below 0 °C were observed almost every day from late December to early February. The temperatures began to increase after that, and days with temperatures below 0 °C were rare in mid-March (Fig. 2a). Consistent with these temperature changes, the observed *Eucalyptus* leaf QY levels began to decrease in mid-December and did not begin to recover until late March (Fig. 2b). The leaf IL levels were clearly increased from early to late February and had not recovered at the end of March (Fig. 2c). Visual observation showed that leaves at the branch tips were completely wilted from late January to mid-February (Fig. S1). Experientially, browning and curling of leaves due to cold damage were visually apparent at the site from the end of January onwards (data not shown). Thus, the timing of this decreasing trend in QY was also synchronous, with almost no delay, with the timing of the daily minimum temperatures that were increasingly below 0 °C. Although the changes in QY levels were drastic, IL changes were seen at the same time or after, when leaves wilted (Fig. S1). These results suggested that QY observation is a highly sensitive index of the damage caused by a decrease in temperature.

### Description of QY observations with meteorological data by a linear regression model

**Figure 3:**
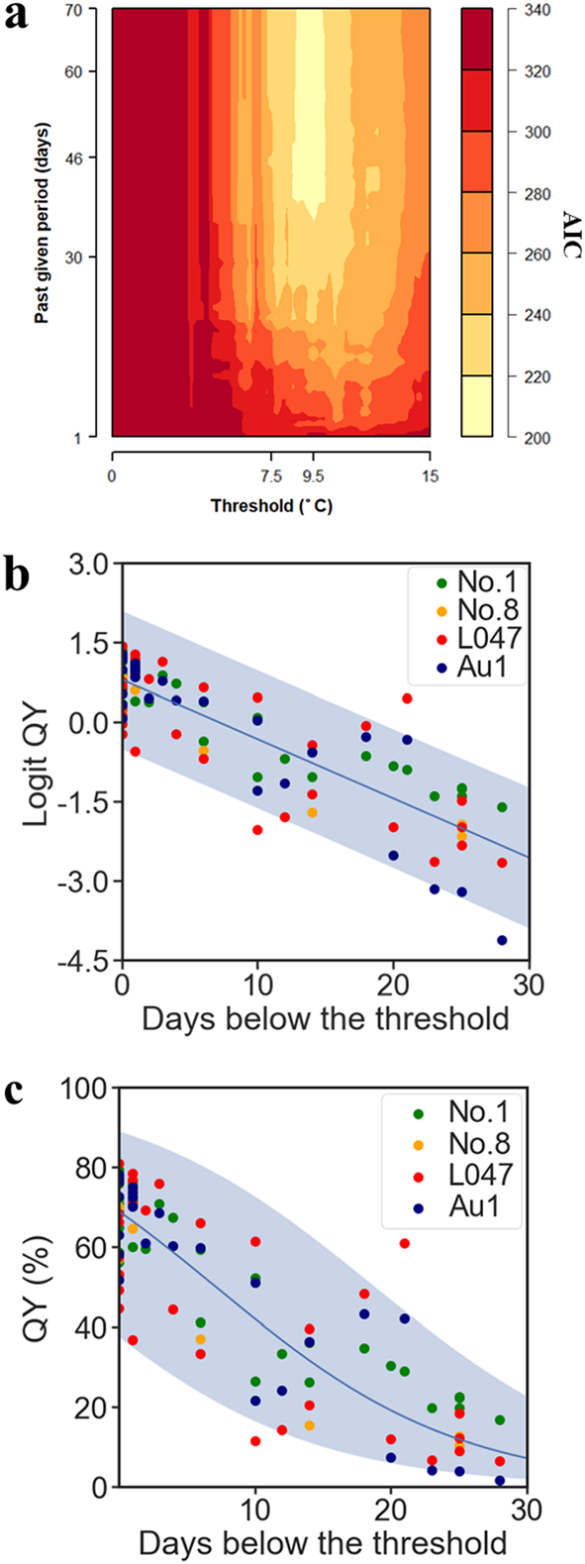
Chilling-unit model for the regression of QY levels during winter (**a**) Distribution of AIC of models using modified chilling accumulation units (days) by daily maximum temperatures. AIC was calculated for a total of 5758 combinations of thresholds (T) and past given periods (L), respectively, and are displayed in a heatmap (see Fig. S2 for other modified type and temperature sort combinations). Observed QYs were best explained by the chilling units given by the combination of T = 9.5 and L = 46. (**b**) The regression line is given by the minimum AIC parameters. The blue solid line and the colored range indicate the regression fit lines and their 95% confidence intervals, respectively. Green, orange, red, and navy dots indicate the observed values of QY (see Fig. S3 for other combination results).

**Figure 4:**
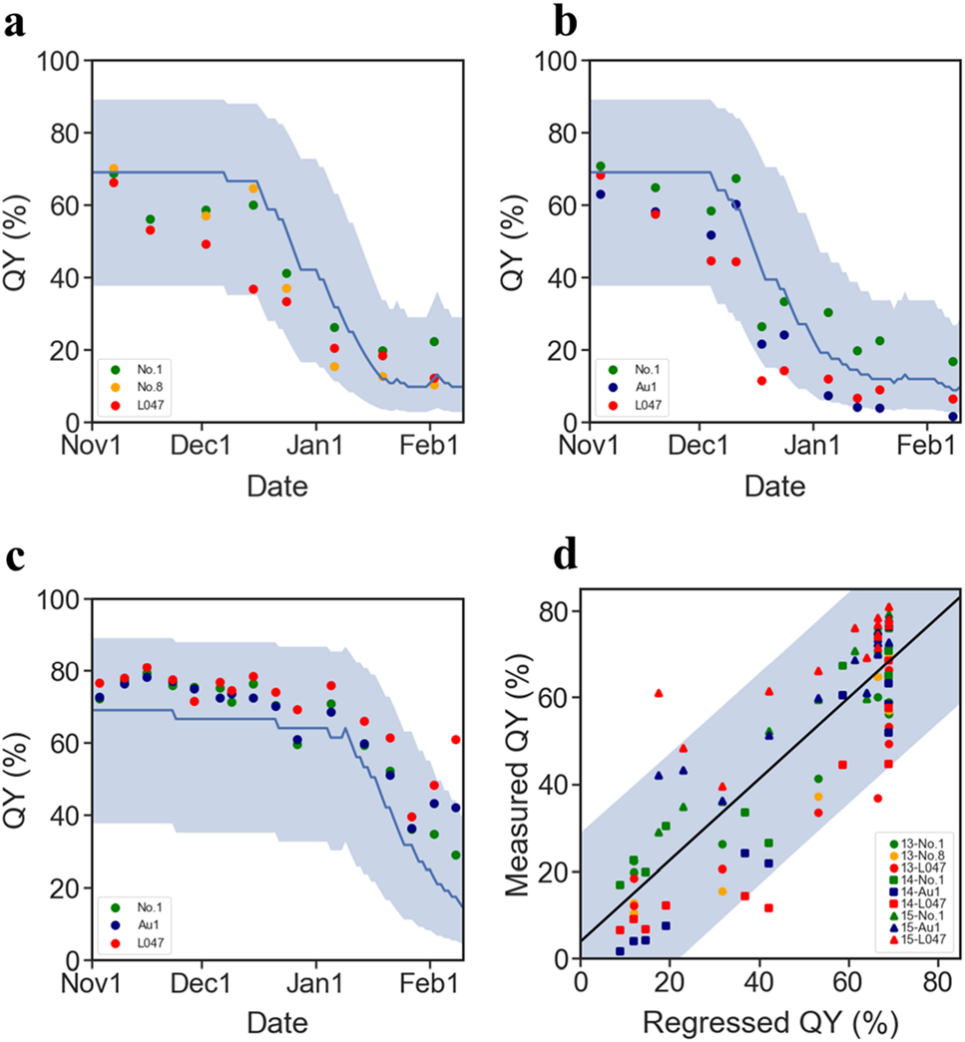
Validation by regression to the training data subsets. (**a**–**c**) Time series of autoregression for the 2013–2014, 2014–2015, and 2015–2016 seasons are shown in (**a**–**c**), respectively. The blue solid line and the colored range indicate regression fit curves and their 95% confidence intervals, respectively. Green, orange, red, and navy dots indicate the observed values of QY. (**d**) Correlation analysis between regression by the model and observed values. Scatter plot of predicted values from the model vs. observed values. Solid lines indicate the regression curves of the plots (see Fig. S4).

**Figure 5:**
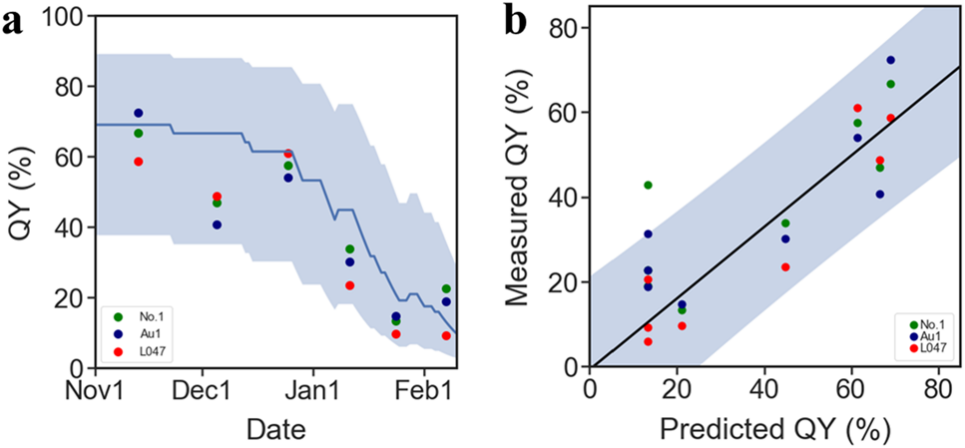
Validation by prediction to the verification data subset. (**a**) Time series of predictions of the model for the 2016–2017 season dataset that was not used for the modeling. The blue solid line and the colored range indicate regression fit curves and their 95% confidence intervals, respectively. Green, red, and navy dots indicate the observed values of QY. (**b**) Correlation analysis between predictions by the model and observed values. The scatter plot shows predicted values from the model vs. observed values. Solid lines indicate the regression curves of the plots.

To explain the relationship between QY and temperature, we prepared 6 types of chilling accumulation units (CUs) from the publicly available meteorological data as explanatory factors in the linear regression model (Fig. S2). Linear regressions were performed among the 25,620 combinations of chilling accumulation units and QY observations, and the maximum likelihood linear regression model was selected by comparing the linear regression results (Fig. 3a and Fig. S5). We calculated Akaike's Information Criterion (AIC) for each of the 25,620 regression models tested using three data subsets used as training data for the model development, i.e., the 2013–2014, 2014–2015, and 2015–2016 subsets (Figs. S3, S4). Comparison among all the regression models showed that the lowest AIC was given from the model using CU_d_max, L=46, Th=9.5_ as explanatory values (Figs. 3a, S5, Table 1). To evaluate the efficiency of this model, we performed regression analyses using the three data subsets that were earlier used to train the model (Fig. S2). Three seasons of data used as training data were divided by year and regressed values by the model were compared with observed values (Fig. 4). Comparing the observed values and range of regression-predicted values by each of three season, clear negative and positive biases were observed between observed values and the regresses values in 2013–14 and 2015–16, respectively (Fig. 4a and c). However, the results of the regression analyses showed that 98 out of a total 102 regressions were plotted within the 95% confidence intervals of the regressions of the model (Fig. 3bc, Fig. 4). The correlation coefficient and coefficient of determination of the regression between the predictions and observed values were 0.87 and 0.75, respectively, and these were the highest among all the models examined (Table 1 and Fig. 4d). Next, we evaluated the model by means of a prediction analysis using the validation data subset for 2016–2017, which was not used to build the model (Fig. S2). The results of the prediction analysis showed that 17 out of a total 18 predictions were plotted within the 95% confidence intervals of the prediction of the model (Fig. 5), and the correlation coefficient and coefficient of determination of the model prediction between the predictions and observed values were 0.84 and 0.70 (Fig. 5b). These results suggested that it would be possible to explain about 70% of the variation of leave QY in *E. globulus* by the number of days with a daily maximum temperature under 9.5 °C in the past 46 days.

**Table 1:** Summary of the likelihoods of models based on a combination of explanatory variables.

CU type	Temp. sort	Temp. threshold (Th) (°C)	Period (L) (days)	AIC	Log lik	Coefficient of determination (R^2^) between predictions and observations
	minimum	−1.25	50	227.2	−111	0.69
	maximum	11.00	41	220.6	−107	0.70
	average	5.00	50	221.0	−108	0.68
	minimum	−4.00	39	213.6	−104	0.73
	maximum	9.50	46	206.5	−100	0.75
	average	2.75	56	209.3	−102	0.72

### Global geographical simulation using publicly available meteorological observation data

**Figure 6:**
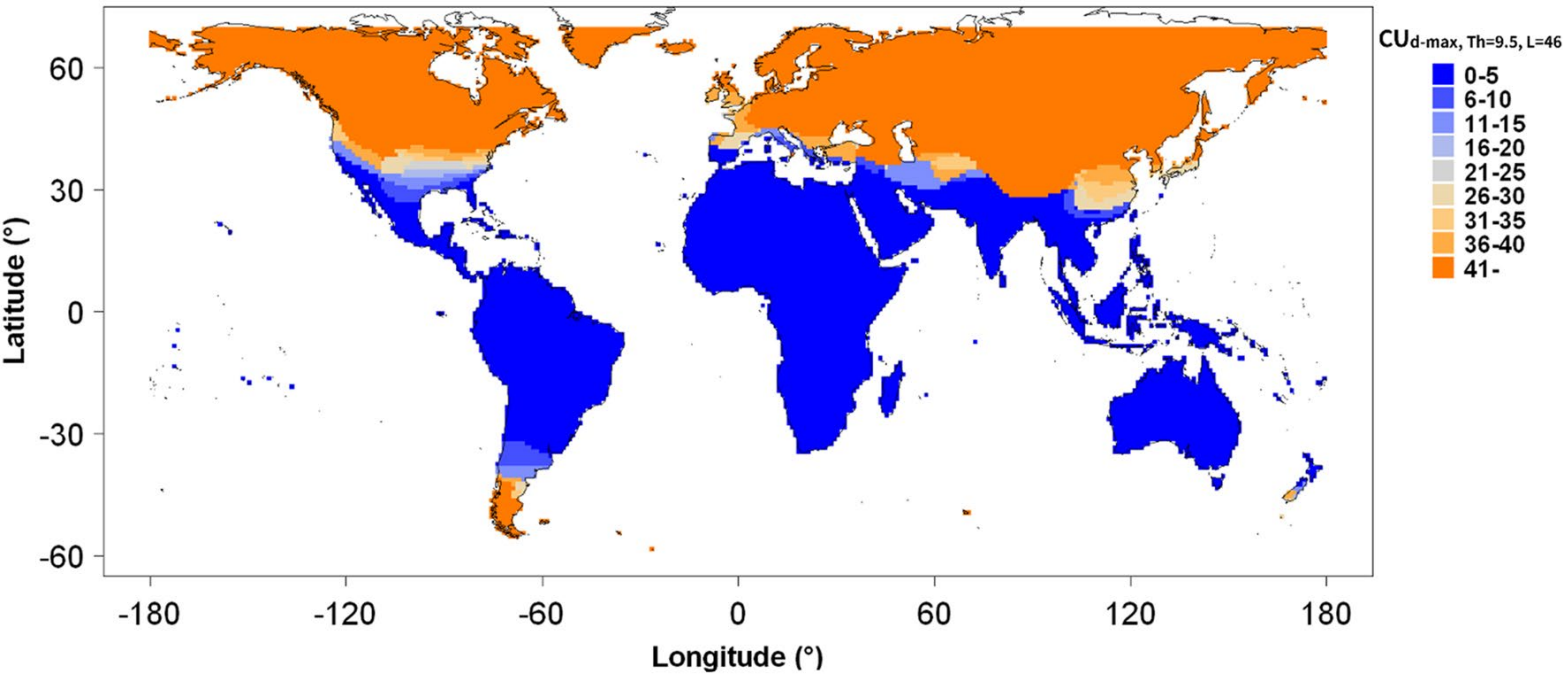
Predicting global *Eucalyptus* plantation potential using calculated chilling units. Using world temperature data from January 2018 to March 2022 and the period maximum CU (i.e., CU_d-max, Th=9.5, L=46_) at about 4500 locations around the world were calculated. CUs at other grids were predicted by the SVM algorithm. Grid were color-coded according to predicted CU values shown on the right side of the panel. The based map was drawn with ‘map’ package of R software under GPL2 licenses.

Using the model developed above, it would be possible to predict the low temperature-induced damage to *E. globulus* trees by means of temperature information alone. By combining this model with machine learning techniques, we considered that it would be possible to predict the QY value of *E. globulus* trees in winter at different cultivation locations around the world (Fig. S6). The requisite global meteorological data were accessible via the internet, and we collected temperature data from over 9,000 locations around the world. Figure 6 shows the distribution of the calculated maximum CU_d_max, Th=9.5, L=46_ values. The distribution of the areas where the CU value were blow 35 overlapped more than 70% with the global *Eucalyptus* plantation forests map developed with information from the Food and Agriculture Organization of the United Nations (FAO-Forestry) (Fig. S7). At a CU value of 35, the QY predicted by the model was 14.8% (confidence interval ranged from 9.4 to 22.0%), which was inferred to be the threshold for the possibility of *Eucalyptus* plantation. On the other hand, the contributions of precipitation and cultivation site elevation to the rate limitation of genus *Eucalyptus* plantations are also known^20^, which allowed us to evaluate the accuracy of this simulation. We concluded that these simulation based on the model would provide a fairly rough estimate of the potential for establishing plantation-based cultivations of *E. globulus*.

### Simulation using meteorological observation data collected over the past 70 years in Japan

**Figure 7:**
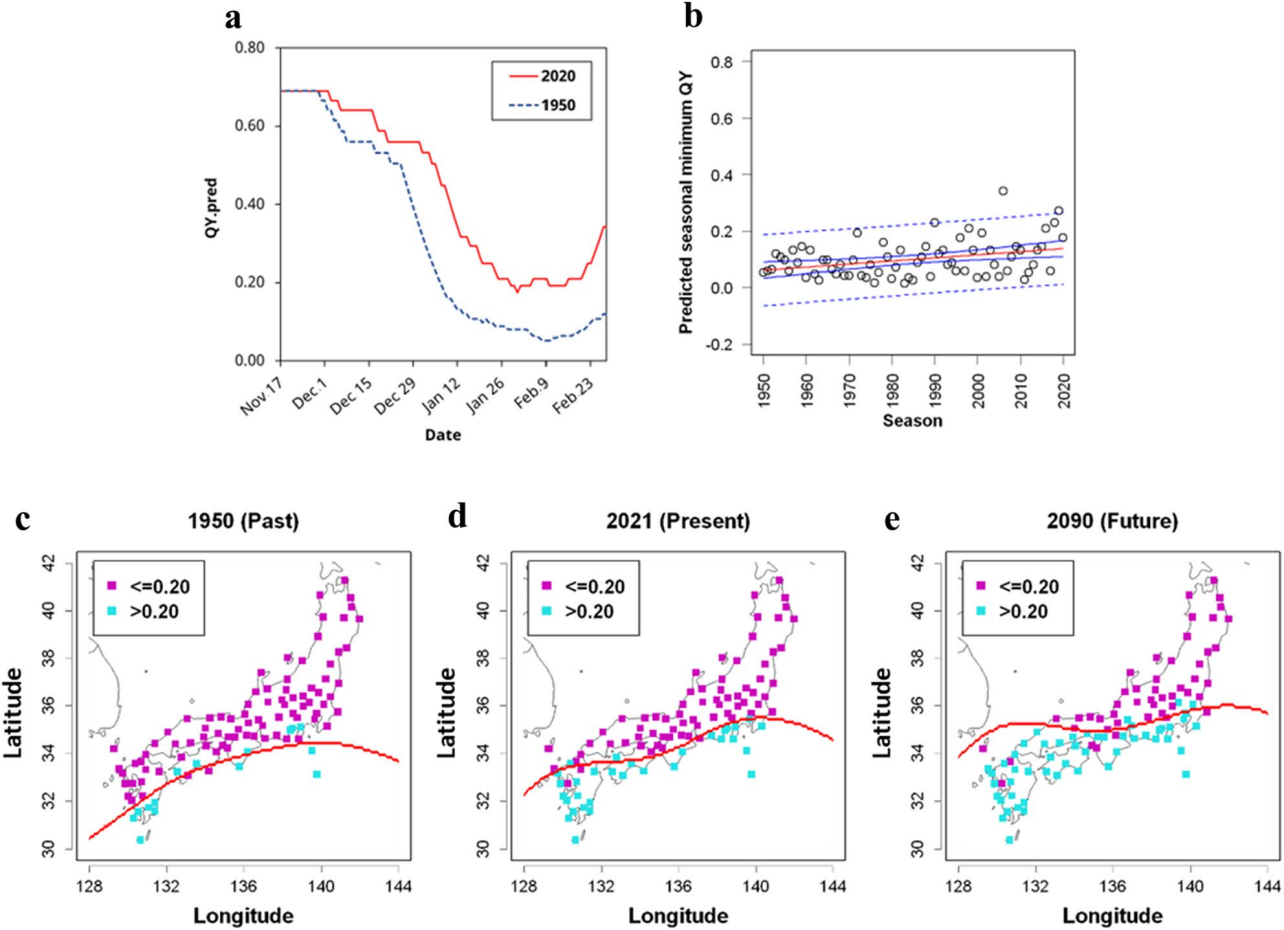
Simulation using meteorological observation data of JMA (**a**) Simulated QYs from late November to February in the 1950 and 2020 seasons. The blue dashed line and red line indicate simulated values for the 1950 and 2020 seasons, respectively. (**b**) Simulation of seasonal minimum QYs in Tsukuba from 1950 to 2020. Open plots indicate seasonal minimum QYs predicted by the established model. The red line, blue lines, and blue dashed lines indicate linear regression lines, 95% confidence intervals, and 95% prediction intervals, respectively. (**c**–**e**) Simulations of the seasonal minimum in 1950, 2021, 2090 were shown in (**c**–**e**), respectively. The red and blue plots show the predicted QY seasonal minimums below 0.20 and above 0.20, respectively. Red lines indicated decision boundaries calculated by the SVM model. The based map was drawn with ‘map’ package of R software under GPL2 licenses.

Next, we provide an example of a simulation focusing on vectors in the time axis direction (Fig. S8). We obtained from the JMA the temperature data for the period of October 1950 through February 1951 and the same months in 2021–2022, and conducted a simulation of QY levels using the model (Fig. 7a). The simulated annual minimum QY levels were 0.05 and 0.19 for 1950/51 and 2021/221, respectively, and the annual minimum levels increased by about 0.01 per decade (Fig. 7a, b). Simulations were also performed in the same manner for 97 major JMA posts around Japan, excluding Hokkaido and Okinawa, and linear regression analyses of the time-series changes in the simulated annual minimum QYs were performed for each post. Using the linear regression models, the annual minimum QYs were regressed for 97 sites in 1950/51, 2020/21, and 2090/91 (Fig. 7c–e). Then, based on the predicted values for the 97 observation sites, the minimum QY for each of approximately 3,000 grids of the land areas of Japan ranging from 30° to 40° N and from 128° to 144° E with a grid size of 0.05° of latitude and longitude was estimated by a machine-learning approach using SVM methodology. The number of sites where the predicted annual minimum QY exceeded 0.20 was calculated to be 33 in 2021 and 50 in 2090, compared to 14 out of 97 locations in 1950 (Fig. 7c–e). These simulations suggest that global warming has increased the potential *Eucalyptus* plantation areas in Japan by about 2.4 times over the past 70 years and will do so by about 1.5 times the 2021 value over the next 70 years.

## Discussion

Although the global area of potential *Eucalyptus* tree plantations is known to be restricted by low winter temperatures, there have been no global predictions of total possible plantation areas. On the other hand, precise plant physiological experiments of large-scale woody plants are complicated and difficult to implement on a large scale. In this study, we therefore tried to express the relationship between winter temperature and low-temperature damage to *E. globulus* trees as a formula by combining observational data obtained in relatively small-scale experimental fields and available meteorological data using statistical methodology.

We chose QY as the quantitative indicator of plant damage caused by cold temperature stress. Although gene expression levels or morphological changes can also be used to quantify cold damage caused to plants, QY is much easier to measure than gene expression levels, and it allows for a more subtle quantification and evaluation of the degree of damage compared to survival rates and the like. In addition, QY can be measured nondestructively without removing the leaves from the tree. Based on these characteristics, QY was adopted in this study as an indicator of low-temperature damage to *E. globulus* trees under open field conditions. In other studies, QY has been used as quantitative indicators for plant damage by biotic and abiotic stresses^18,21,22^.

To convert the chilling accumulation unit of the temperature data applied in this study, we used the methodology of Akikawa et al*.* which was proposed to explain the annual expression of the *FLC* gene in a natural population of perennial *Arabidopsis halleri*^17^. In this study, we prepared a total of 25,620 chilling accumulation units, tested the linear regression model between the logit transformed QY observed values, and selected one model by maximum likelihood estimation. The maximum likelihood model was the model using CU_d_max, Th=9.5, L=46_ as explanatory variables; this model explains QY values by number of days that the daily maximum temperature was below 9.5 °C over the past 46 days. These results suggest that the main cause of low-temperature damage in *E. globulus* leaves is not physical, i.e., that damage is not caused by freezing water, but rather is due to physiological functions induced by low temperatures. For example, it was possible that the function of the active oxygen scavenging system in photosynthesis is reduced due to the reduced fluidity of the thylakoid membrane cortex due to low temperatures^23^.

The main disadvantage of the current model is that the confidence intervals are relatively wide (Figs. 3b, c, 4a–c). By increasing the size of the training dataset used for the model development, the size of the confidence intervals would be expected to decline. The recruitment of environmental data other than temperature might also improve the prediction accuracy of the model. On the other hand, it is difficult to prepare additional plant physiological observation data to input for the model development. In the future, it may be necessary to develop a new model building method that can utilize remote sensing data such as the normalized difference vegetation index (NDVI) using drones and satellites.

Meteorological data observed around the world, including temperature data, have been shared and made available worldwide through the Japan Meteorological Agency (JMA). Thus, based on the prediction by a model developed using the observation data from numerous sites, the possibilities of establishing an* E. globulus* plantation for each of approximately 14,600 grids of the world's land area, with a grid size of 2° of latitude and longitude, were estimated by a machine learning approach using supported vector machine (SVM) methodology. The predicted range of *E. globulus* plantation potential obtained based on the predicted QY, using meteorological data from various parts of the world, was generally consistent with the northern and southern borders of the map of genus *Eucalyptus* plantation areas prepared based on the Global Biodiversity Information Facility (GBIF)^20^ and other data (Figs. 1a, 6). This suggested that the model can be extended to locations other than those used in the model. It was a limitation that this model uses only temperature data as an explanatory variable, whereas other models commonly consider not only temperature but also rainfall, solar radiation, and geology to predict the growing area. Nonetheless, it was interesting to note that this model was able to make relatively reasonable predictions using only temperature data. Possible reasons for this were that this study focused only on cold damage and the fact that we were able to monitor the damage in an area that is the northernmost area of *Eucalyptus* growth. As a result, our model behaves as a predictor of whether a site was a suitable northern borderline environment for *Eucalyptus* forestry plantation. It made sense to think of the model as predicting the northern and southern borders rather than the growth area. Evidence of this was that our model shows a high potential for *Eucalyptus* growth in the Sahara Desert and in the Middle East. This was a result that does not consider factors such as precipitation at all. However, because the model focused on only one trait, it was very easy to develop the model, collect the data and understand the results. Thus, an evaluation based on a single trait has its own advantages and makes a unique contribution.

Using the developed model, we attempted to verify changes in low-temperature damage due to global warming in Japan (Fig. 7a–e). Future QY was predicted from climate predictions based on rates of temperature increase between previously recorded and present temperature data. Although there were differences from year to year, the data from 1950 to 2020 show a trend of increasing temperature in many parts of Japan. The annual increase in average daily maximum temperature during winter, i.e., from October to the end of February, at 101 sites around Japan, including the 97 sites used in the prediction, is 0.016 °C, which was consistent with Intergovernmental Panel on Climate Change (IPCC) estimates^24^. If this degree of increase were to continue until 2090, the annual minimum QY increase could be estimated. It was suggested that the degree of winter damage may be alleviated, and the northern borders of potential *Eucalyptus* plantation areas might be extended, expanding the *Eucalyptus* growing range.

Our results indicated that this model has potential reliability based on a fairly limited set of experimental observations. On the other hand, the confidence interval of the prediction was wide, and at the present stage, there are many problems in using it as a practical tool for predicting the feasibility of reforestation. It was a well-known fact that E. globulus plantation trees derived from warm climates, are vulnerable to low temperatures, however it was not described about the multi-year seasonal observation of cold damage of *E. globulus*. The model was novel and established by combining publicly available meteorological data and the quantitative physiological data obtained by ourselves. As one of the uses of the model, we are now making plans to apply this simulation model to an evaluation of the transgenic *E. globulus*. Since it was labor-intensive to conduct large-scale field trials of transgenic plants, especially woody plants at the research level in various regions, using this statistical model evaluation was worth considering. The use of statistical models to evaluate transgenic plants allowed us to extend the results from limited data, and a more detailed evaluation is expected. Such methods would be a tool to assist developers of small-scale transgenic crops/plants who cannot conduct experiments on a large scale and would facilitate the development of transgenic plants. We are currently conducting the development of transgenic *E. globulus* harboring a gene related to fatty acid desaturation, and we would like to utilize simulations using this model as an example of evaluation of the transgenic *E. globulus* in the near future.

## Conclusion

In this study, we proposed a novel statistical model to explain some of the damage caused by cold temperature stress on *E. globulus* based on experimental observation data collected from an experimental field trial. The simulation using this model suggested that it was possible to some extent to predict cold injury that occurs at times and locations different from the data provided for model building. The prediction intervals for this model's predictions were wide and there is still room for improvement. On the other hand, it was expected that the earlier screening evaluation of cold-tolerant tree breeding will lead to a significant reduction in the time and cost involved in breeding forest trees.

## Materials and methods

### Plant materials

Four clones of *Eucalyptus globulus* Labill., i.e., No. 1, No. 8, Au1, and L047, were developed within Nippon Paper Industries (Tokyo) as its proprietary resources in accordance with the relevant guidelines and regulations, and were licensed from the company to the University of Tsukuba for research purposes, including for use in the present investigation^25,26^. The cloned plants were derived from the bulked seeds for industrial plantation use, and were not wild plants^24^. The sterile seedlings were acclimatized in a cultivation room for about 1 week, then transplanted into pyramidal pots with a mixture of equal proportions of Kanuma soil, Akadama soil, and Hyuga soil, and acclimatized in the cultivation room for another 3 weeks. After acclimation, the plants were moved to a specific netting room and allowed to acclimatize for about 1 month. After acclimation, the plants were moved to a specific netting room and acclimatized for about 1 month, after which they were transplanted to the field with an appropriate amount of Magamp K medium grain N-P-K-Mg = 6-40-6-15 (Hyponex Japan, Osaka, Japan). In this study, we tested two lines planted in November 2011 (4 plants for No. 1; 2 plants for No. 8), one line planted in October 2012 (2 plants for L047), and three lines planted in October 2013 (15 plants for No. 1; 5 plants for Au1; 5 plants for L047) (Fig. S3).

### Field trial conditions

The field trial was performed in an isolated field at the Gene Research Center, Tsukuba Plant Innovation Research Center, University of Tsukuba, from October 2011 to September 2017. This field is located in Tsukuba, in the Northern Kanto Plain, and at the center of the main island of Japan (36^o^07′ N, 140^o^06′ E) (Fig. 1a). The Köppen climate classification for this site was temperate humid (Cwa), with an average daily temperature of 3.1 °C in winter (January, average of 1990–2020) (Fig. 1b). This area is considered one of the northernmost reaches of possible *Eucalyptus* plantations (Fig. 1a). The plantlets were arranged in a 1 m grid (Fig. 1c, d).

### Physiological datasets used for analyses

The 6-year field trials for the evaluation of *Eucalyptus* trees’ responsiveness to low temperatures were conducted from 2011 to 2017, and QY monitoring data were recorded over four winter seasons. To evaluate the relationship between fluctuations of *E. globulus* leaf QY levels and air temperature, we tried to seek linear regression models using meteorological observation data to predict the variability of QY. In this study, the first three seasons, i.e., 2013–2014, 2014–2015, and 2015–2016, of the four-season subset of QY observations were used as training data for model development and selection, and the last season, 2016–2017, was used as test data for model evaluation (Fig. S3).

### Measurement of chlorophyll fluorescence quantum yield

The photosynthetic quantum yield (QY) was measured as an indicator of plant damage at low temperatures. QY was measured using a FluorPen-FP100 (Photon System Instruments, Drasov, Czech Republic). Three leaves at the tips of side branches derived from the seventh pair of leaves from the top of the stem were selected and QY was measured approximately every two weeks from November to March in the 2013–2014 winter, and from November to March in the other winters (Fig. S6).

### Measurement of ion leakage rate

The ion leakage rate (IL) was measured as an indicator of cell membrane damage in *E. globulus* trees planted in 2011 and 2012, from November 2013 to June 2013, in parallel with the QY measurements on the same leaves. Three leaf discs of 7 mm in diameter were punched from the sampled leaves using a cork borer and suspended in 1.6 mL of MilliQ water in a 2 mL disposable tube. The tubes were then placed in a mixing rotor (TR-350; AS ONE Co., Osaka, Japan) and agitated at room temperature for 2 to 3 h. After stirring, 40 μL of the suspension was collected from each tube and the initial conductance values were measured by a conductance meter (LAQUA twin COND B-771; HORIBA, Kyoto, Japan). The tubes were then heated at 90 °C for 30 min using a drying oven (MOV-212; Sanyo Electric Co., Osaka, Japan), set in the tube rotator again, and stirred for 2 to 3 h at room temperature. After stirring, 40 μL of the suspension was collected from each tube, and the post-conductance values were measured using the conductance meter. Ion leakages (%) were calculated by dividing the initial value by the post-conductance values.

### Meteorological data

The temperature data used were the daily maximum temperature, daily minimum temperature, and daily average temperature at 1.6 m above the ground, as measured and published by the CRiED^27^. Daily climate data for the past 70 years for various regions of Japan were taken from historical weather data distributed by the Japan Meteorological Agency (JMA) of the Ministry of Land, Infrastructure, Transport, and Tourism of Japan. The global temperature data used in the growth area prediction were obtained from the ClimatView database, which was compiled from the World Meteorological Organization’s source data by JMA. We used the maximum temperature at each of 4599 locations and the minimum temperature at each of 5152 locations measured between January 1st, 2018 and March 29th, 2022; each location had a missing data rate of 10% or less. Temperature on days with missing data were supplemented with the arithmetic mean of the measurements immediately before and after the day.

### Modeling approach

In this study, we developed the following linear regression model of QY values of leaves of *E. globulus* in the field explained by the chilling accumulation units (CUs):

$${\text{logit }}\left( {{\text{QY}}} \right) \, = {\text{ a }}*{\text{ CUs }} + {\text{ b}}$$

We examined two approaches for calculating the chilling accumulation units. In the first, the chilling accumulation units by temperature (CU_t_) were defined as the sum of the temperature below a certain threshold temperature (Th) within a certain period of days (L). The chilling accumulation units by day (CU_d_) were defined as the sum of the number of days below the threshold temperature (Th) within a certain period for accumulation (L). Both types of chilling units were calculated for three statistical values of temperature parameters—i.e., daily maximum (mx), daily minimum (mi), and daily average (av))—and thus a total of six types of chilling units (CU_t_mx_, CUt_mi, CU_t_av_, CU_d_mx_, CU_d_mi_, and CU_d_av_) were designated. Each kind of CUs were calculated with a total 4,270 combinations of the threshold temperatures (Th) in the 15 °C range by 0.25 °C increments (61 sorts) and the period for accumulation (L) in the range from 1 to 70 days by 1-day increments (70 sorts); the total number of UCs calculated was 25,620. The temperature ranges (Th) for the three temperature parameters are shown in Table S1.

We calculated AICs of 25,620 sorts of linear regression using three training data subsets—i.e., three data subsets of 2013–2014, 2014–2015, and 2015–2016—and the model that showed the lowest AIC was determined as the maximum likelihood model. CUs were calculated using the temperature data obtained from the CRiED as described in the previous section, and a and b in the formula and AIC were calculated by the "glm" function of R software (version 4.1.3). The heat maps in Figs. 3A and S2 were drawn by the "contour" function of R software. R software was used under GPL2 license.

### Model evaluation

We compared and verified the observed value and the regression value from the most likely model from the three data subsets used as the training data for the model development, i.e., the 2012–2013, 2013–2014, and 2014–2015 subsets. Furthermore, we compared and verified the observed values and the estimated values from the most likely model from the validation data subset that was not used for model determination, i.e., the 2015–2016 subset (Fig. S2).

### Geographical simulation analysis

The global temperature data were obtained from the Global Weather Data Tool database provided by the JMA. The daily maximum and minimum temperature data for about 4 years from January 1st, 2018 to March 29th, 2022 at 9,148 (max) and 9,482 (min) observation sites were obtained, and the data at 4,599 (max) and 5,152 (min) sites that each had a missing data rate of 10% or less were used for the simulation. The daily predicted QYs were calculated for each of the sites using the obtained data, and the predicted annual minimum QYs were gathered for each observation site. Next, the predicted season minimum QYs for locations other than the observation sites were calculated by a machine learning approach using the SVM method for geographical grids with sector dimensions of 2° of latitude and longitude. The potential plantation availabilities were determined by setting annual minimum QY thresholds adopted from the distribution of actual global *Eucalyptus* plantation areas for the predicted annual minimum QYs.

### Global warming simulation

Daily climate data collected over the past 70 years in various regions of Japan were used to simulate changes in habitat due to global warming. Daily temperature data of 101 sites for the past approximately 70 years (1950–2021) were obtained from JMA web site, and used to calculate the predicted QY for each location and each year. Next, the equation of the linear regression between the time series and the predicted QYs were calculated for each site. The third step was to calculate the predicted QYs for 2090 at each site. And finally, the QY values of the 101 sites were converted into binary values categorized as above or below the threshold of 0.4. Based on these values, the binary values for each of about 3000 grids obtained by gridding the land area of Japan in units of latitude and longitude of 0.1° were calculated by the supported vector machine (SVM) algorithm.

SMV was implemented with the ksvm function of the kernlab package (version 0.9-31) of R software. The blank maps were drawn with the "map" package (version 3.4.1), and the binary boundaries were drawn with the contour function of R software. R software, kernlab package, and map package were used under GPL2 licenses.

## Supplementary Information

Supplementary Information.

## Data Availability

The datasets used and/or analyzed during the current study are available from the corresponding author on reasonable request.
