# Supplementary material for: A statistical modeling approach based on the small-scale field trial and meteorological data for preliminary prediction of the impact of low temperature on Eucalyptus globulus trees
Source: Sci Rep. 2023 Jun 22;13:10138. doi: 10.1038/s41598-023-37038-8 (PMC10287712; doi:10.1038/s41598-023-37038-8)
Supplement: Supplementary file 1 — Supplementary Information. [file 41598_2023_37038_MOESM1_ESM.docx]

**Supplementary Information**


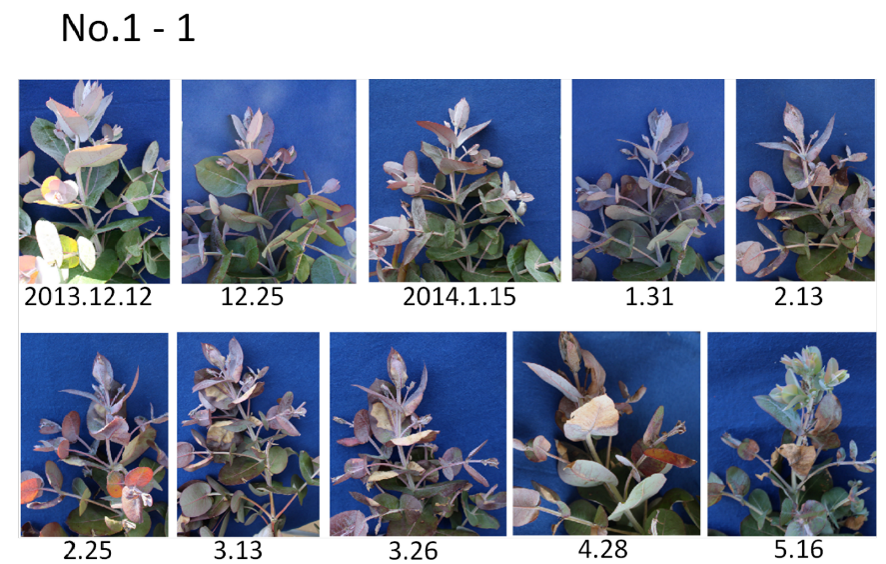


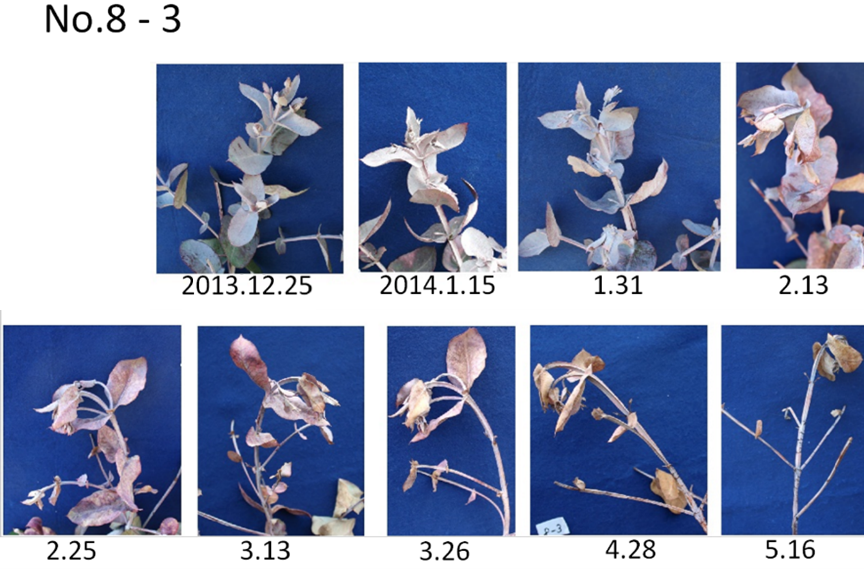


**Fig. S1. Visual observation of changes in *E. globulus* levels during winter (reference material)**

The photos were taken blanches of a respective individual tree of line of No.1 and No.8 from December 2013 to May 2014. In the No.8 line, browning and curling of leaves were observed from end of January onwards, and the damaged leaves did not grow back in the sea.

In the N0.1 line, a change in leaves color at the branch tips was observed, but no significant damage was observed by visual in this year.
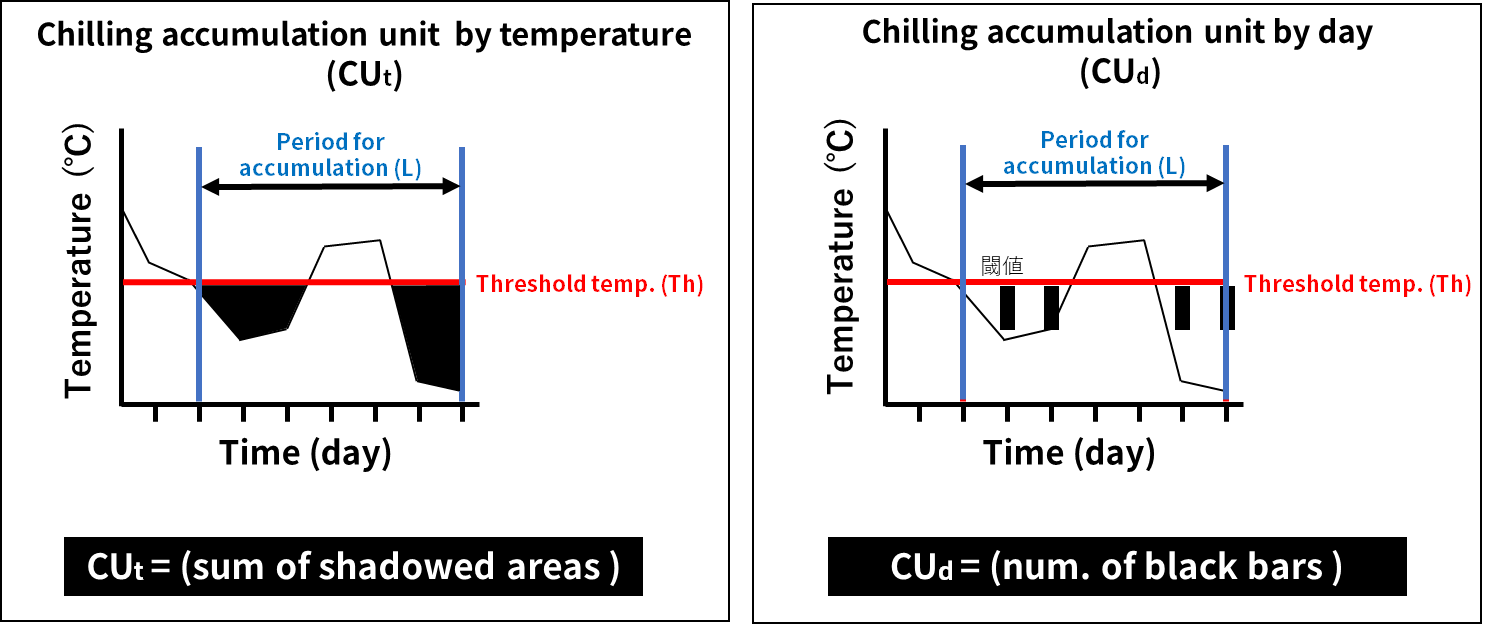


**Fig. S2 Schematic of the chilling accumulation units used in this study.**

Two types of chilling accumulation units, i.e., chilling accumulation units by temperature (CU_t_) and chilling accumulation units by day (CU_d_), were used in this study for the calculate leave QYs of field grown *E. globulus*. CU_t_s were given as the sum of the temperature below a certain threshold temperature (Th) within a certain period of days (L). CU_d_s were given as the sum of the number of days below the threshold temperature (Th) within a certain period for accumulation (L). Both type of chilling units were calculated for three statistical values of temperature parameters, i.e. the daily maximum (mx), daily minimum (mi), and daily average (av)), and thus a total of six kinds of chilling units (CU_t_mx_, CUt_mi, CU_t_av_, CU_d_mx_, CU_d_mi_, and CU_d_av_) were designated.


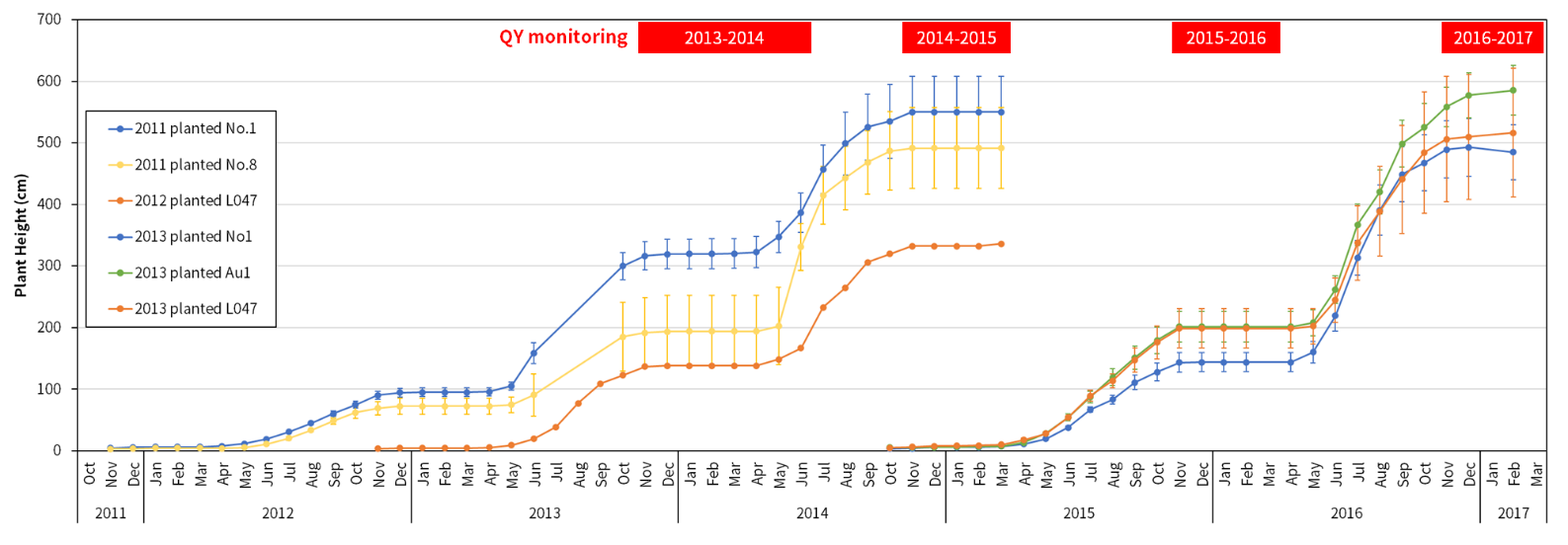


**Fig. S3. Growth of *E. globulus* trees used in this study.**

In this study, we tested 2 lines planted in November 2011 (4 plants for No. 1; 2 plants for No. 8), one line planted in October 2012 (2 plants for L047), and 3 lines planted in October 2013 (15 plants for No. 1; 5 plants for Au1; 5 plants for L047). Each plot and error is indicated as the mean and standard error, respectively. The upper red box indicates the QY observation period used for model creation and validation.


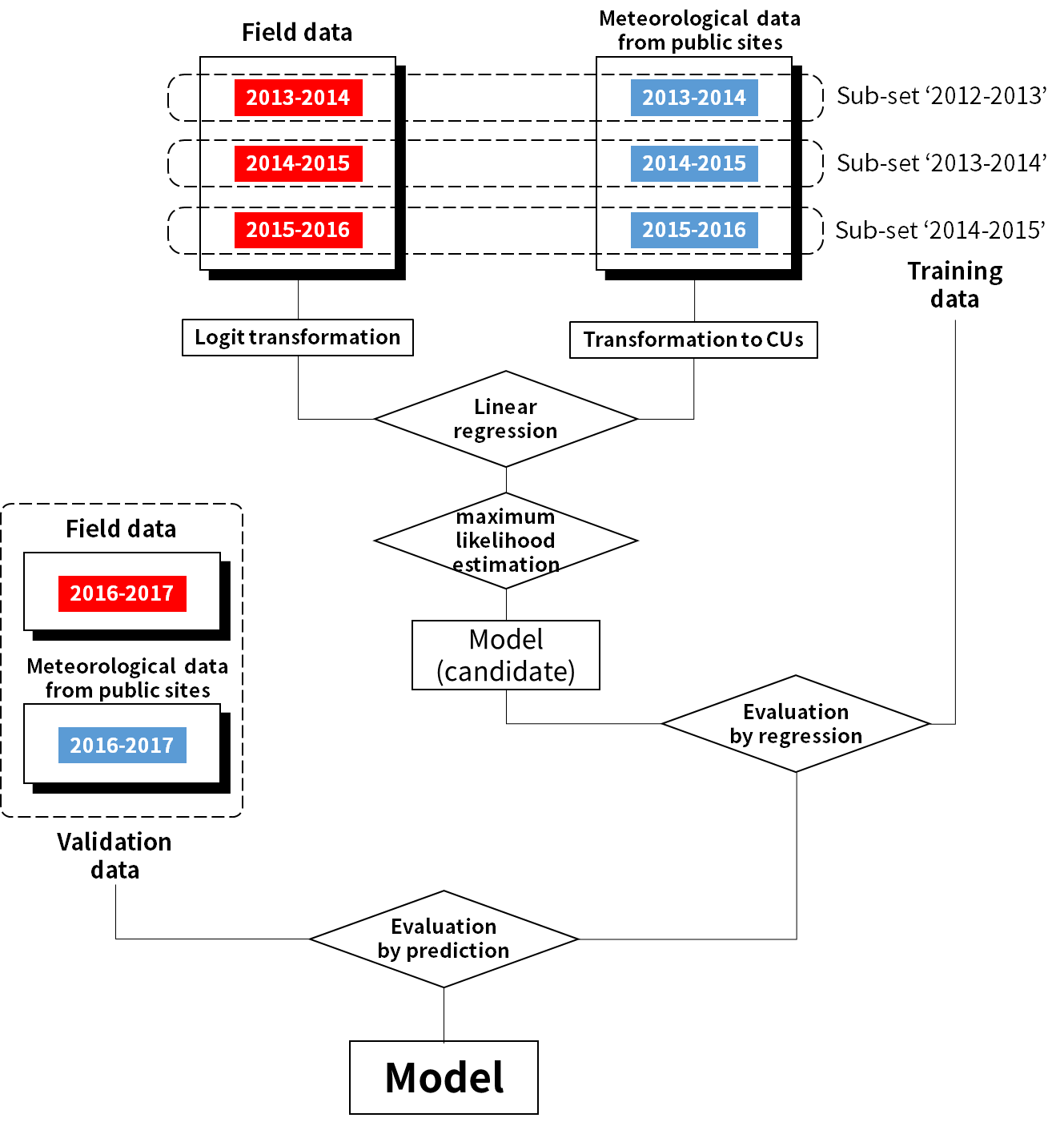


**Fig. S4 Flowchart of the model development.**

Three data subsets (2013–2014, 2014–2015, 2015–2016) were used for the regression model developments. QY and temperature values were transformed to logit values and chilling accumulation values, respectively, and linear regression was performed. After calculation of all the possible combinations of the model, the most reasonable model was selected by the maximum likelihood estimation. The model was first evaluated by regression analyses to the training data, and then by prediction to the validation data.

See the subsection “Modeling approach” in the Materials and Methods.


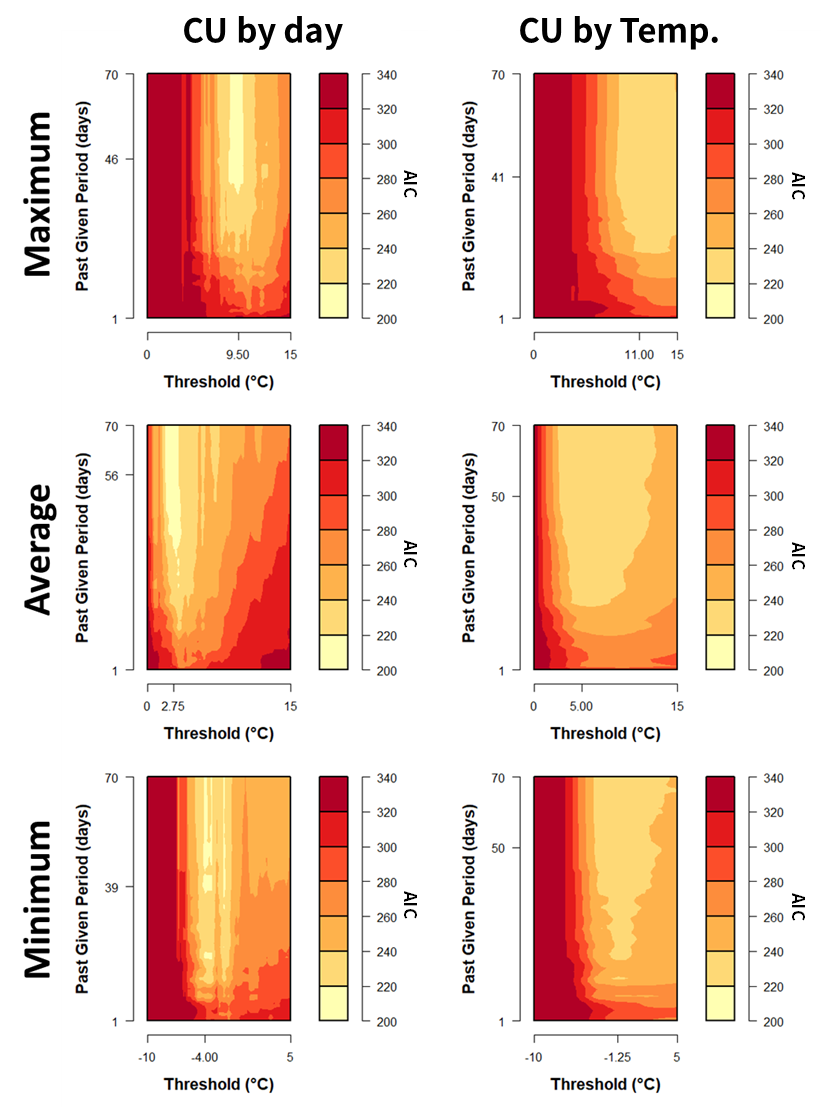


**Fig. S5. Distribution of AICs of models given from models using CU_t_s and CU_d_s as the explain factors.**

Distribution of AIC of models using chilling-unit all combination. AICs were calculated for a total of 25,620 combinations of thresholds (Th) and past given periods (L), for each temperature accumulation type (CU_t_) and day accumulation type (CU_d_). The left and right panels display the distribution of AICs as heatmaps given from models using CU_t_s and CU_d_s as the explanatory factors, respectively.


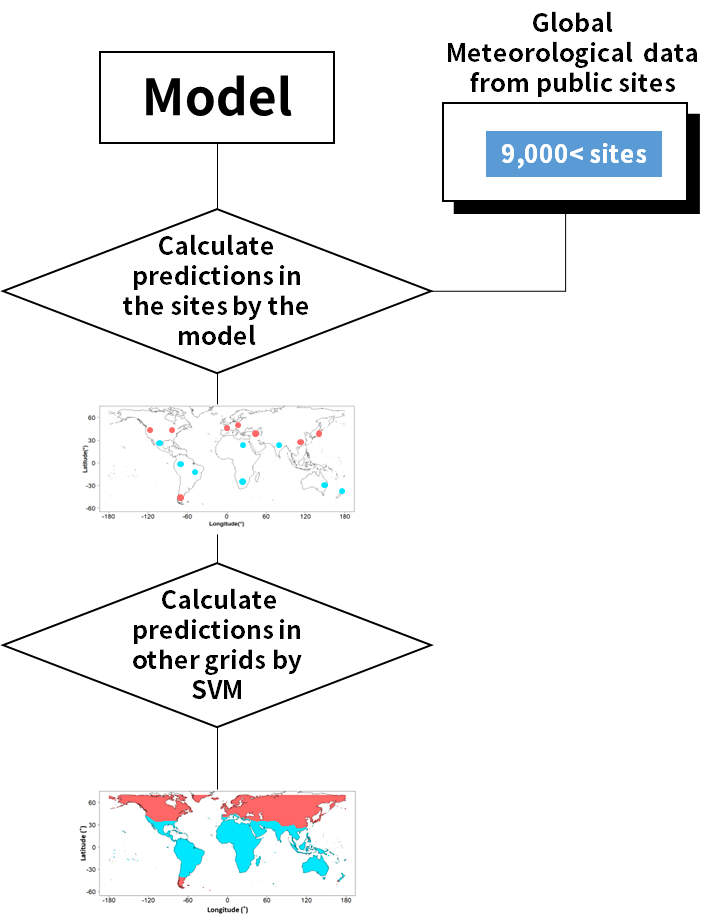


**Fig. S6 Scheme flow chat of Geographical simulation analysis**

The temperature data on over 9,000 locations were collected via the Global Weather Data Tool database provided by the JMA. The annual smallest QYs for the locations were predict by the model. Using the predictions, that of other locations were calculated by a machine learning approach using the SVM method for geographical grids with sector dimensions of 2° of latitude and longitude.

See subsection “Geographical simulation analysis” in Materials and Methods. The based maps were drawn with ‘map’ package (version 3.4.1) of R software (version 4.1.3) under GPL2 licenses.

**
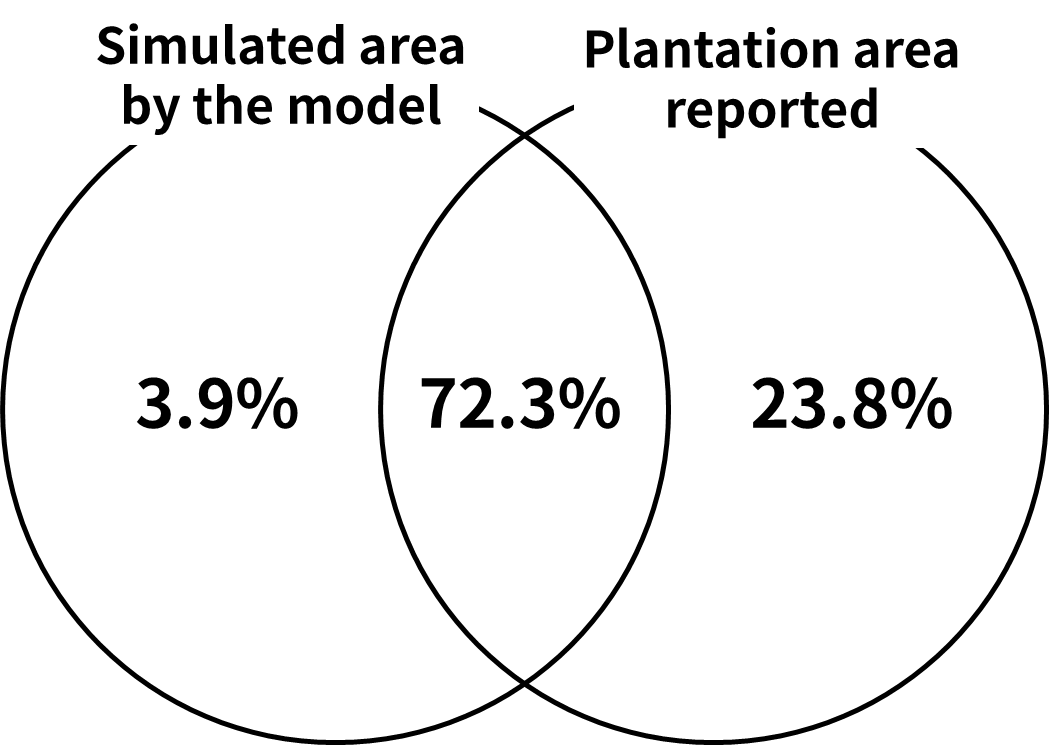
**

**Fig.S7 Comparison of the potential *E. globulus* plantations predicted by the model and the global *Eucalyptus* plantations map.**

The Venn diagram showed proportions of over-lapped area and non-over-lapped aeras, which were calculated by comparison between the potential *E. globulus* plantations predicted by the model (sky blue area in Figure 6) and the global *Eucalyptus* plantations map based on the previous report (shown in green or blue in Figure 1a).


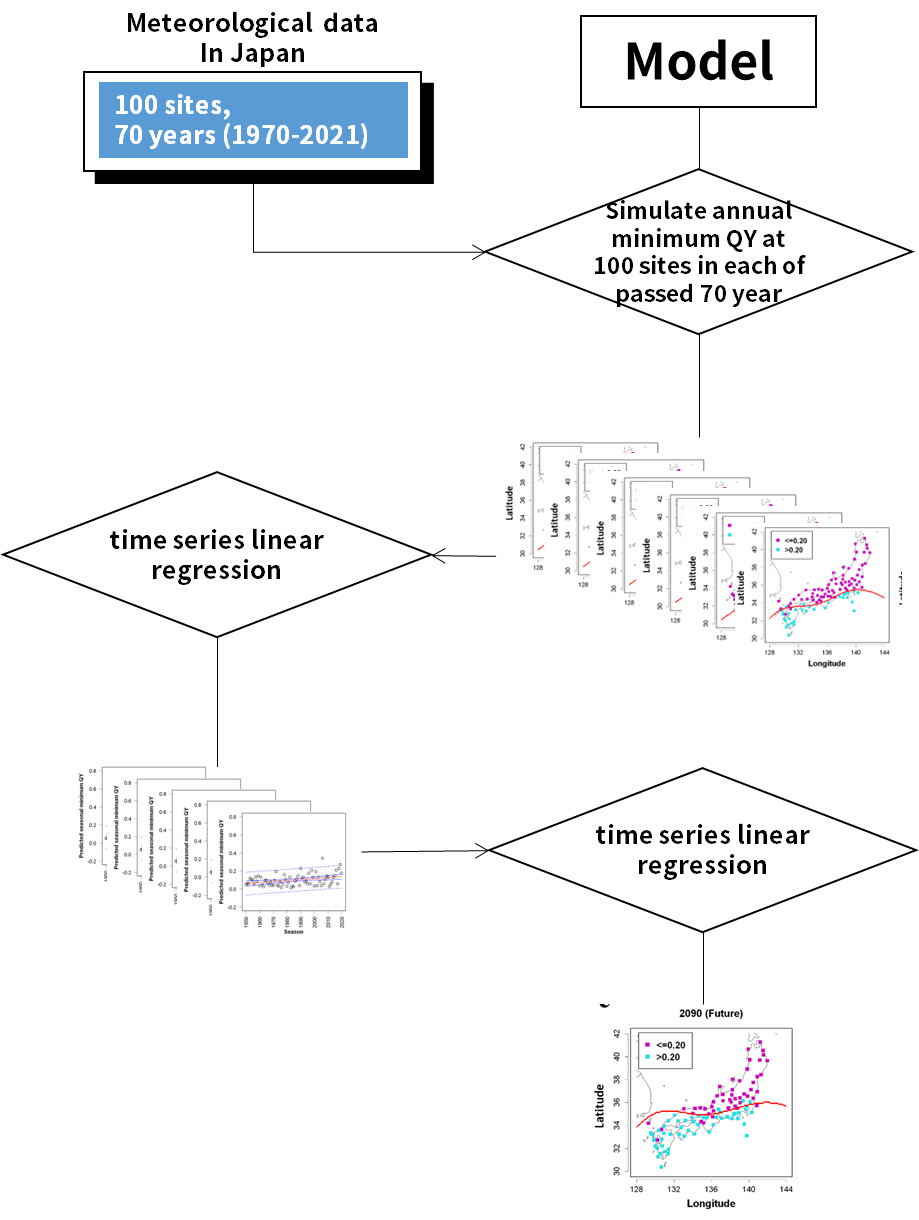


**Fig.S8 Scheme flow chat of Global warming simulation in Japan**

Daily climate data in various parts of Japan for the past 70 years, were used to simulate changes in habitat due to global warming. Daily temperature data of 101 sites for the past about 70 years (1950–2021) were obtained from JMA web site, and predicted QY for each location and each year, was calculated respectively. Next step, the equation of the linear regression between the time series and the predicted QYs was calculated for each site. The third step, the predicted QYs in the 2090 were calculated for each site, respectively. The fourth step, QY values of 101 sites were converted into the binary values categorized by above or below the threshold of 0.4. Based on these values, the binary values for each about 3000 grids obtained by gridding the land area of Japan in units of latitude and longitude of 0.1° were calculated by the supported vector machine (SVM) algorithm.

See subsection “Global warming simulation” in Materials and Methods.

**Table S1 Temperature ranges (Th) for different temperature parameters using calculation of CUs**

| **Temperature parameter** | **Minimum of range** | **Maximum of range** |
| --- | --- | --- |
| Daily maximum | 0°C | 15°C |
| Daily minimum | -10°C | 5°C |
| Daily average | 0°C | 15°C |
